# Supplementary material for: Trends in Antimicrobial Resistance Patterns in Neisseria Gonorrhoeae in Australia and New Zealand: A Meta-analysis and Systematic Review
Source: Antibiotics (Basel). 2019 Oct 23;8(4):191. doi: 10.3390/antibiotics8040191 (PMC6963718; doi:10.3390/antibiotics8040191)
Supplement: Supplementary file 1 [file antibiotics-08-00191-s001.zip › Figure Legend.docx]

Figure Legend:

Figure 1: PRISMA flow diagram

Figure 2: Meta-regression plots showing the odds ratios for changes in proportion and weighted average proportions of isolates susceptible to each antibiotic over time in Australia, Australian States and Territories and New Zealand.

Figure 3: Meta-regression plots showing the odds ratios for changes in proportion and weighted average proportions of isolates with decreased susceptibility to each antibiotic over time in Australia, Australian States and Territories and New Zealand.

Figure 4: Meta-regression plots showing the odds ratios for changes in proportion and weighted average proportions of isolates resistant to each antibiotic over time in Australia, Australian States and Territories and New Zealand.

Figure S1: Meta-regression plots showing the weighted average proportion of isolates that were penicillinase producing *N. gonorrhoeae* (PPNG) over time in Australia, Australian States and Territories and New Zealand.

Figure S2: Meta-regression plots showing the weighted average proportion of isolates that demonstrated chromosomally mediated resistance (CMRP) over time in Australia, Australian States and Territories and New Zealand.

Tables and Supplementary Tables

Table 1: Summary of the main characteristics of included studies and quality assessment scores

Table S1: Summary of the data extracted on the number of isolates tested, number of isolates susceptible, having decreased susceptibility and resistance from each study that was included in the meta-analysis.

Table S2: Proportion of *N. gonorrhoea* isolates susceptible, decreased susceptible and resistant to various antimicrobials and the Odds ratio for changes in rates in Australia, Australian States and Territories and New Zealand over time.
