# Supplementary figures and images for: Trends in Antimicrobial Resistance Patterns in Neisseria Gonorrhoeae in Australia and New Zealand: A Meta-analysis and Systematic Review
Source: Antibiotics (Basel). 2019 Oct 23;8(4):191. doi: 10.3390/antibiotics8040191 (PMC6963718; doi:10.3390/antibiotics8040191)

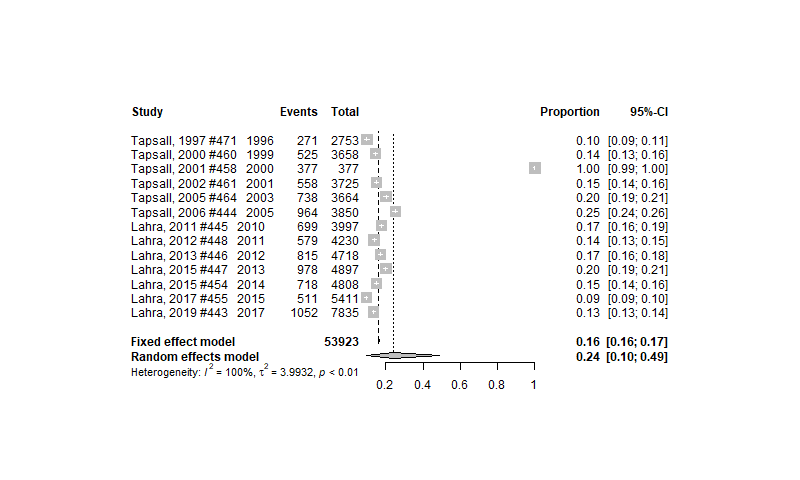

Supplement: Supplementary file 1 [file antibiotics-08-00191-s001.zip › Attachment 4_Figure S2_AUSTRALIA CMRP.png]

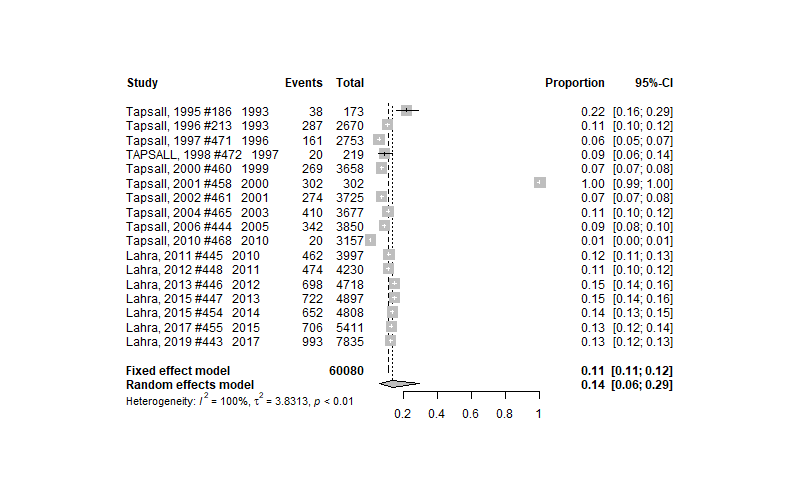

Supplement: Supplementary file 1 [file antibiotics-08-00191-s001.zip › Attachment 3_Figure S1_AUSTRALIA PPNG.png]
